# Hippocampal corticotropin-releasing hormone neurons support recognition memory and modulate hippocampal excitability

**DOI:** 10.1371/journal.pone.0191363

**Published:** 2018-01-18

**Authors:** Andrew Hooper, Patrick M. Fuller, Jamie Maguire

**Affiliations:** 1 Tufts University School of Medicine, Department of Neuroscience, Boston, Massachusetts; 2 Beth Israel Deaconess Medical Center, Department of Neurology, Harvard Medical School, Boston, Massachusetts; University Paris Diderot, FRANCE

## Abstract

Corticotropin-releasing hormone (CRH) signaling in the hippocampus has been established to be important for mediating the effects of stress on learning and memory. Given our laboratory’s recent characterization of a subset of hippocampal CRH neurons as a novel class of GABAergic interneurons, we hypothesized that these local GABAergic hippocampal CRH neurons may influence hippocampal function. Here we applied an array of molecular tools to selectively label and manipulate hippocampal CRH neurons in mice, in order to assess this interneuron population’s impact on hippocampus-dependent behaviors and hippocampal network excitability. Genetically-targeted ablation of hippocampal CRH neurons *in vivo* impaired object recognition memory and substantially enhanced the severity of kainic acid-induced seizures. Conversely, selective activation of CRH neurons *in vitro* suppressed the excitability of the mossy fiber-CA3 pathway. Additional experiments are needed to reconcile the functions of GABA and CRH signaling of hippocampal CRH neurons on hippocampal function. However, our results indicate that this interneuron population plays an important role in maintaining adaptive network excitability, and provide a specific circuit-level mechanism for this role.

## Introduction

The hippocampus is critical for spatial navigation and episodic memory formation [1, 2]. Both of these tasks require precisely timed synchronous output, which is accomplished by over 20 distinct classes of hippocampal GABAergic interneurons [3, 4]. Furthermore, this must be done while clamping the overall excitability and synchrony of the hippocampus within an adaptive range, in order to avoid runaway excitation lead to an epileptic seizure, a pathological state of hypersynchronous hyperexcitability to which the hippocampus is particularly vulnerable [5, 6]. In order to better understand how interneurons accomplish this extremely delicate and dynamic balancing act, it is essential that we identify and thoroughly characterize hippocampal interneuron types. Each type is defined by a unique combination of characteristics, including its location within the hippocampus, molecular markers, firing patterns, and the subcellular localization of its synaptic contacts with pyramidal neurons [3]. Additionally, interneurons play a critical role in generating network-wide oscillations, and each interneuron type tends to fire only at specific phases of a given oscillation [7, 8]. For example, the extensively-studied basket cells are found in *stratum pyramidale*, express parvalbumin, and form contacts primarily on the cell bodies and proximal dendrites of pyramidal neurons; they can sustain rapid firing of action potentials, and tend to fire specifically on the descending phase of theta oscillations [9]. Taken together, the intrinsic properties and functional connectivity of an interneuron class provide valuable insight into their functions within the network such as the encoding of place fields, and pathogenic mechanisms for diseases such as epilepsy [10, 11]. Now, thanks to the development of cell type-specific genetic tools, we can selectively image and control the activity of individual interneuron types in mice. By assessing the impact of these manipulations on local network activity, hippocampus-dependent behavior, and pathological activity such as epileptic seizures, we can then begin to develop realistic models bridging the single unit, the local network, and higher-order brain functions and states.

We recently characterized the connectivity and intrinsic molecular and electrophysiological properties of a novel hippocampal interneuron type: the back-projecting corticotropin-releasing hormone (CRH) interneuron [12]. This unique class of hippocampal interneurons projects from CA1 *stratum pyramidale* to form extensive GABAergic synapses on CA3 principal cells, suggesting an important role in providing feedback inhibition to control the excitability of the trisynaptic circuit. However, to date functional studies of hippocampal CRH neurons have focused solely on the neuropeptide CRH itself, missing the potential impact of their synaptic connectivity [13, 14]. In the present study, we expand on our initial characterization by probing CRH interneurons’ relevance to hippocampus-dependent processes such as emotional behavior and recognition memory, as well as their impact on seizures in a model of temporal lobe epilepsy. Finally, we propose a circuit-level mechanism for these functional effects, based on back-projection CRH interneurons’ impact on the excitability of the mossy fiber-CA3 pathway.

## Materials and methods

### Animals

Adult (60–90 days old) mice were housed at the Tufts University School of Medicine, Division of Laboratory Animal Medicine, in clear plastic cages (four mice/cage) in a temperature- and humidity-controlled environment with a 12 h light/dark cycle (lights on at 0700 h) and *ad libitum* access to food and water. Only male mice were used in field potential recording experiments; in all other experiments, approximately equal numbers of male and female mice were used. All procedures were approved by the Tufts University Institutional Animal Care and Use Committee and adhered to the ethical guidelines presented in the National Institutes of Health Guide for the Care and Use of Laboratory Animals [15].

Transgenic mice expressing Cre recombinase under control of the CRH promotor (BAC CRH-Cre) were obtained from the Mutant Mouse Regional Resource Center (MMRRC) and have been described previously [16]. Knock-in mice expressing Cre recombinase under control of the endogenous CRH promotor (CRH-ires-Cre) were obtained from the Jackson Laboratory (Stock #012704). Mice expressing tdTomato specifically in CRH neurons (CRH-Ai9) were generated by crossing either BAC CRH-Cre or CRH-ires-Cre mice with floxed Ai9 mice (Jackson Lab, Stock #007909). Mice expressing tdTomato and Channelrhodopsin specifically in CRH neurons (ChR/CRH) were generated by crossing BAC CRH-Cre mice with floxed Channelrhodopsin mice (Jackson Laboratory, Stock #017455).

### Virus injection and EEG surgery

Mice were anesthetized by intraperitoneal (i.p.) injection of a ketamine-xylazine mixture (100 mg/kg ketamine, 10 mg/kg xylazine). AAV2-GFP (Vector BioLabs #7004), AAV8-hSyn-DIO-hM3D(G_q_)-mCherry (UNC Vectore Core), AAV8-hSyn-DIO-hM4D(G_i_)-mCherry (UNC Vector Core), or AAV-FLEX-DTA (*CMV-β-globin-DIO-mCherry-DTA-hGH pA* generated by Dr. Patrick M. Fuller, Harvard Medical School) was stereotaxically injected into the dorsal hippocampus of each hemisphere (500 nL; posterior 2.0mm, lateral 1.5mm, depth 1.5mm) and the syringe was left in place for 5 minutes before withdrawal. For the EEG studies, mice were outfitted with an EEG/EMG headmount (Pinnacle #8201) utilizing stainless steel screws as two EEG leads, a reference electrode, EMG leads, and an animal ground. All animals were treated with buprenorphine (0.5mg/kg) preoperatively and then administered as needed to alleviate pain and/or distress to the animals during the three day postoperative observation period. Posthoc analysis was performed for each experiment to validate targeting and verify expected manipulations.

### Immunohistochemistry

Mice were anesthetized by isoflurane inhalation, decapitated, and their brains were rapidly removed and fixed by immersion fixation in 4% paraformaldehyde overnight at 4°C. For CRH immunostaining, mice were instead transcardially perfused with phosphate-buffered saline and 4% paraformaldehyde before immersion fixation to improve staining quality. Brains were cryoprotected in 10% and 30% sucrose in phosphate-buffered saline, rapidly frozen in isopentane on dry ice, and stored at -80°C until 40 μm coronal sections were collected using a Leica cryostat. Sections were incubated with an antibody against CRH (1:10,000, rabbit, provided by Dr. Paul Sawchenko on behalf of Dr. Wylie Vale) or c-fos (1:10,000, rabbit, Calbiochem), followed by biotinylated anti-rabbit antibody (1:200, Vector Laboratories) and streptavidin AlexaFluor 488 conjugate (1:1,000, Molecular Probes). Images were collected on a Nikon A1R confocal microscope and analyzed using ImageJ.

### Patch clamp recording

To confirm chemogenetic modulation of CRH neurons, whole-cell patch clamp recordings were performed on visually identified CRH neurons expressing G_i_ DREADD or AAV-GFP in hippocampal coronal slices (350 μm). An internal solution containing (in mM): 130 K-gluconate, 10 KCl, 4 NaCl, 10 HEPES, 0.1 EGTA, 2 Mg-ATP, 0.3 Na-GTP (pH = 7.25, 280–290 mOsm) and electrodes with DC resistance of 5–8 MΩ were used to record changes in the firing rate and resting membrane potential (RMP) following bath application of CNO (500nM). The RMP was measured over a 100 ms, action potential-free period immediately before application of CNO and compared to the RMP after stabilization following CNO administration. Series resistance and whole-cell capacitance were monitored throughout the recording period and slices were excluded from the dataset if these values changed by >20% during the experiment.

### Field potential recording

Transverse hippocampal sections (400 μm) were collected from adult male ChR/CRH mice and Cre^-/-^ littermates. First, mice were anesthetized by isoflurane inhalation, decapitated, and their brains rapidly removed into ice-cold sucrose cutting solution containing (in mM): 3 KCl, 87 NaCl, 7 MgCl_2_-6H_2_O, 1.25 NaH_2_PO_4_, 0.5 CaCl_2_-2H_2_O, 50 sucrose, 25 dextrose, and 25 NaHCO_3_. Sections were then cut using a Leica vibratome and incubated at 36°C in normal artificial cerebrospinal fluid (nACSF) containing (in mM) 126 NaCl, 2.5 KCl, 2 MgCl_2_-6H_2_O, 1.25 NaH_2_PO_4_, 2 CaCl_2_-2H_2_O, 10 dextrose, 1.5 sodium pyruvate, 1 L-glutamine, and 26 NaHCO3. Sections were incubated for at least one hour before being transferred to the recording chamber, which was maintained at 34°C by an in-line heater (Warner Instruments) and perfused continuously at a rate of 6 mL/minute with nACSF throughout the experiment. All solutions were continuously bubbled with 95% O_2_ and 5% CO_2_.

Evoked field potentials from the mossy fiber/associative commissural fiber-CA3 pathway were recorded using a DP-311 differential amplifier (Warner Instruments) and LabChart 7 data acquisition software (AD Instruments). A bipolar tungsten electrode was used to deliver stimuli and a micropipette made of borosilicate glass (World Precision Instruments) with DC resistance of 5–8 MΩ, backfilled with nACSF and its tip gently broken off to reduce series resistance and improve sensitivity, was used for extracellular field recordings. Postsynaptic field excitatory postsynaptic potentials (fEPSPs) were evoked by stimulating over *stratum radiatum* of CA3 at the border of the hilus and the responses were recorded in *stratum radiatum* of CA3 at least 500 μm from the stimulating electrode. After a threshold stimulation intensity was determined by recording a threshold response at a width (W) of 60 μs with no response at 20 μs, input-output curves were generated by increasing the stimulus pulse width from 20 to 240 μsec in 20 μsec steps, applying four stimuli per pulse width at 0.1 Hz. The maximum slope of the responses (volts per second) was measured over a 0.5–1 ms window of the fEPSP rising phase and the average slope was calculated at each W used for each recording. Input/output curves were fit with a Boltzmann equation: f(W) = (MAX/(1 + exp((W—W_50_)/k)) + MAX), where W is stimulus width, MAX is the maximum response, k is a slope factor, and W_50_ is the stimulus width that elicits 50% of MAX.

### Behavior testing

Behavioral experiments were run at the same time of day (between 10am and 4pm) in at least three separate cohorts of animals with all experimental groups represented to ensure reproducibility and to control for batch effects. Males and females were used for all experiments. We did not observe a sex difference in any of the behaviors assessed and therefore the data have been collapsed across sex. All behavior experiments were conducted in the Tufts Center for Neuroscience Research, P30 NS047243 (Jackson). Mice were acclimated to the testing facility for one hour before testing. Additionally, DREADD-injected mice and sham- or GFP-injected littermate controls were acclimated to i.p. injection once daily for three days before testing. Clozapine N-oxide (CNO, 10 mg/kg) was administered i.p. to these animals 40 minutes before testing (before acquisition training only, in the fear conditioning and object recognition experiments) to permit peak DREADD activation [17]. AAV-GFP controls were also treated with CNO to assess any off-target effects [18]. All enclosures were cleaned using 70% ethanol between tests to remove odor cues.

#### Open field test

Locomotor activity and anxiety-like behavior were assessed using the open field test [19]. Each mouse was placed in the center of a 40cm x 40cm open field enclosure with 16 x 16 equally spaced photocells (Hamilton-Kinder). The total number of beam breaks and the distance traveled and time spent in the center of the enclosure were measured for 10 min using MotorMonitor software (Hamilton-Kinder).

#### Light/dark box test

The light/dark box test was also used to assess anxiety-like behavior, as it is generally considered more sensitive than the open field test in this regard [19]. Each mouse was placed in the open, illuminated compartment of a 22cm x 43cm light/dark box enclosure with 4 x 8 equally spaced photocells (Hamilton-Kinder). The total number of beam breaks and the distance traveled and time spent in the light compartment of the enclosure were measured for 10 min using MotorMonitor software.

#### Forced swim test

The forced swim test was employed to evaluate depression-like behavior [20]. Each mouse was placed into a cylinder (21cm diameter) containing approximately 2 L of water at room temperature (22–25°C). Each trial was videotaped, and the latency to immobility and total time spent immobile over 6 min were measured by an investigator blind to the experimental group.

#### Fear conditioning

Hippocampal-dependent and hippocampal-independent learning and memory were assessed by employing contextual and cued fear memory, respectively, in the tone-shock classical fear conditioning paradigm [21–23]. Experiments were conducted at the same time of day training and contextual memory were performed between 10am-12pm and cued retrieval was performed at 3-5pm. For fear memory acquisition, each mouse was placed in a rectangular box with a steel grid floor (Coulbourn Instruments, 12”W x 10”D x 12”H) and allowed to acclimate for 3 min. The mouse was then given two 20 sec tones (2800 Hz, 80 dB) 1 min apart, each of which ended with a 2 sec, 0.7 mA foot shock. Fear memory retrieval was tested the following day. Each mouse was returned to the same rectangular box, and freezing behavior was measured for 3 min in the absence of tone or shock to assess contextual fear memory retrieval. Mice were then returned to their home cage for at least 3 hours before cued fear memory retrieval. In the cued retrieval test, each mouse was placed in a rectangular plastic container with black and white vertical stripes along the sides and bedding scented with 1% acetic acid. In this novel context, each mouse was presented with the same tones delivered during acquisition but no foot shocks. Freezing behavior was measured for a 3 min baseline period, and throughout the 20 sec tones and subsequent 1 min gaps. Freezing behavior was analyzed using Actimetrics FreezeFrame software (Coulbourn Instruments; bout length 1 sec), and percent time freezing was calculated for contextual and cued retrieval [22].

#### Object recognition memory

The object recognition memory test was applied to assess recognition memory. Mice were acclimated to empty bedding-free test chambers (6”W x 10”D x 5.5”H) for 10 min daily for three days before testing. On the first testing day, each mouse was moved into a bedding-free acquisition chamber with two identical objects (stainless steel hex bolts) fixed to the floor at opposite ends of the chamber. The chamber was videotaped for 5 min while mice explored the chamber and became familiar with the objects. Each mouse underwent this familiarization trial three times, with a 15 min interval between trials. After a 3-hour retention interval, each mouse was moved into a bedding-free retrieval chamber with one familiar object (stainless steel hex bolt) and one novel object (cylindrical nylon washer) of similar sizes and fixed on opposite ends of the chamber, and a 5 min exploration session was videotaped. Finally, after a 24-hour retention interval, the retrieval session was repeated using an alternate novel object (rectangular plastic block). All sessions were then evaluated for object preference ratios by an investigator blind to the experimental group. The location of the novel object (left *versus* right end of the chamber), was counterbalanced across experimental groups to control for nonspecific location preference effects, and the left:right ratio from familiarization trials was calculated to quantify any such preference.

### Stress paradigms

Restraint stress was applied by coaxing mice headfirst into 50 mL falcon tubes with air holes cut in the conical tips to allow breathing, and capping the tubes to prevent escape. Acute stress consisted of a single 30 min session, while chronic stress consisted of 14 consecutive daily 30 min sessions. Foot shock stress consisted of the acquisition phase of the tone-shock classical fear conditioning paradigm described above. In all stress paradigms, mice were sacrificed 1 hr after the beginning of the final stressor to capture immediate early gene expression in stress-associated brain regions.

### EEG recording

One week after the end of behavioral testing, EEG recording was performed to quantitatively assess the severity of kainic acid (KA) -induced seizures [24]. EEG recordings were performed using a preamplifier with 100x gain and a 1.0 Hz high-pass filter (Pinnacle Technology, part #8202-SE). LabChart Pro software (ADInstruments) was used for data acquisition and analysis. A 10 min baseline period was recorded prior to an i.p. injection with 10 mg/kg KA dissolved in 0.9% NaCl injection saline and EEG activity was then recorded for an additional 2 hr period. Electrographic seizure activity was identified as an event at least 2.5 times the standard deviation of the baseline signal in amplitude and at least 5 sec in duration, as previously described [25]. Additionally, periods of rhythmic spiking lasting at least 30 sec and clearly distinct from baseline activity in frequency and amplitude were identified by visual inspection; these abnormal electrographic events were considered along with seizure activity collectively as “epileptiform activity” [25–27]. For each animal, the total duration of epileptiform activity was calculated, as well as the average duration of each epileptiform event and the latency from KA injection to the first event. Finally, in order to distinguish between periods of high-frequency (ictal-like) and low-frequency (interictal-like) seizure activity, data was high-pass filtered at 15 Hz and band-pass filtered from 1 to 3 Hz, respectively [27].

### Statistical analysis

All data are reported as mean ± SEM. For object recognition memory data, a one sample t-test was used to evaluate each experimental group’s novel:familiar exploration ratio against a hypothetical mean of 1.00 (i.e. chance). All other behavioral datasets employed a two-way ANOVA to identify main effects of sex, main effects of injected virus, and interactions between the two, followed by Tukey’s honest significant difference (HSD) test for pairwise group comparisons. For c-fos colocalization experiments, one-way ANOVA was employed, followed by Tukey’s HSD test. A Student’s t-test was used for all other experiments to determine significant differences between experimental groups.

## Results

### The BAC CRH-Cre mouse is a useful tool for manipulating a subset of hippocampal CRH neurons

**Fig 1:**
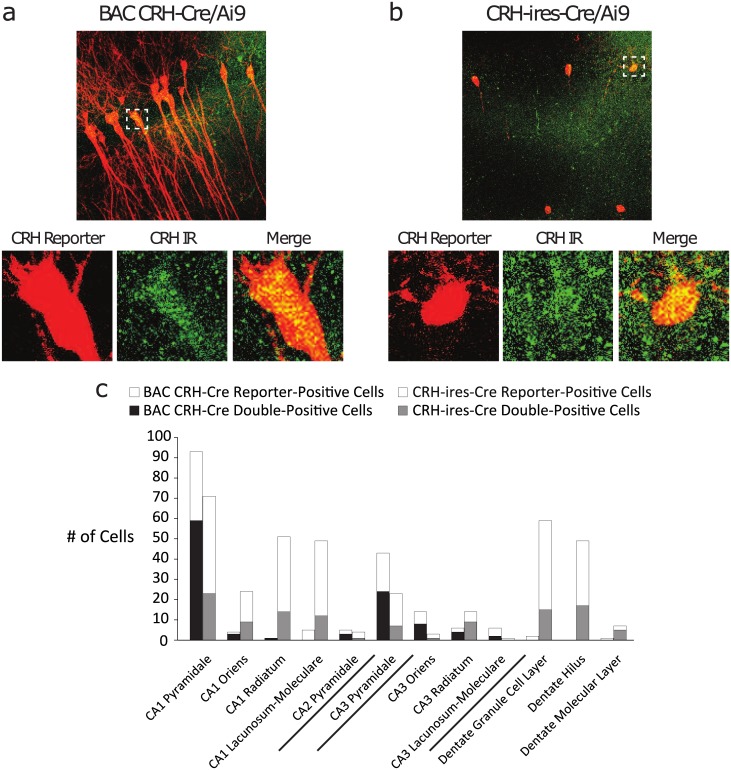
Comparison of hippocampal CRH reporter expression between BAC CRH-Cre and CRH-ires-Cre mouse lines. a, Representative image of CRH reporter expression (red) and CRH peptide immunoreactivity (green) in hippocampal area CA1 from BAC CRH-Cre/Ai9 reporter brain section. Below, example of double-positive neuron. b, Representative image of CRH reporter (red) and immunoreactivity (green) in CA1 from CRH-ires-Cre/Ai9 brain section. Below, example of double-positive neuron. c, The number of cells expressing CRH reporter alone (hollow bars) or double-positive with CRH peptide immunoreactivity (solid bars) was compared between reporter lines for each hippocampal subregion. BAC CRH-Cre and CRH-ires-Cre lines display markedly distinct patterns of CRH reporter expression and reporter-immunoreactivity colocalization. BAC CRH-Cre reporter-positive neurons reside primarily in CA1 stratum pyramidale and display high colocalization.

Multiple mouse lines intended to target Cre recombinase expression specifically to CRH neurons are now available, and variations in the expression patterns among these lines have recently been described [28, 29]. The BAC CRH-Cre mice were generated using a modified bacterial artificial chromosome (BAC) containing Cre under control of the CRH-specific promoter. It is accepted that BAC transgenics often accurrately recapitulate the spatial and temporal pattern of gene expression [30]. The CRH-ires-Cre mice are knock-in mice generated in which Cre recombinase expression is driven by endogenous promoter/enhancer elements [31]. The fidelity of Cre recombinase expression in the BAC CRH-Cre line has recently been questioned [28], although the reported expression pattern does not match our findings [16, 32]. In order to determine whether the BAC CRH-Cre line faithfully targets Cre expression to *bona fide* hippocampal CRH neurons, we assessed CRH reporter (tdTomato) expression and its colocalization with CRH peptide immunoreactivity in BAC CRH-Cre/Ai9 and CRH-ires-Cre/Ai9 hippocampal sections. CRH-ires-Cre/Ai9 sections displayed substantial reporter expression through multiple strata of CA1 and the dentate gyrus, with more limited expression in CA3 (Fig 1b and 1c). In contrast, BAC CRH-Cre/Ai9 reporter was restricted primarily to *stratum pyramidale* of area CA1, with some additional expression in CA3 *stratum pyramidale* and sparse expression in any other subregion (Fig 1a and 1c). In each line, the pattern of reporter expression was consistent between dorsal and ventral aspects of the hippocampus (data not shown). Additionally, BAC CRH-Cre/Ai9 sections displayed considerable colocalization between the reporter and CRH immunoreactivity in *stratum pyramidale* of CA1 (63.4%) and CA3 (55.8%), while CRH-ires-Cre/Ai9 sections showed lower colocalization in these hippocampal subregions (32.4% and 30.4% respectively) (n = 4 matched sections per mouse, 4 mice per experimental group). These results suggest that multiple distinct subpopulations of hippocampal CRH neurons exist, and further indicate that the BAC CRH-Cre line faithfully captures one subpopulation which resides principally in CA1 *stratum pyramidale*, as we previously described [12]. The restricted expression of Cre in the BAC CRH-Cre line enables us to focus specifically on the function of this unique population of hippocampal interneurons for all further experiments.

### Hippocampal CRH neurons exhibit low basal activation and are recruited under hyperexcitable conditions

**Fig 2:**
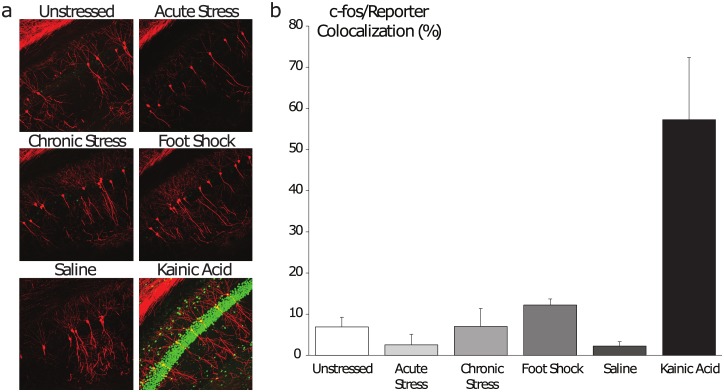
Assessment of hippocampal CRH neurons’ activity across physiological states. a, Representative images of CRH reporter expression (red) and c-fos immunoreactivity (green) in area CA1 of the dorsal hippocampus. b, The ratio of double-positive neurons to total reporter-positive neurons was determined for each state. Hippocampal CRH neurons display low levels of c-fos activation under baseline conditions and following stress, but are highly active following KA-induced seizures.

In an attempt to determine under what conditions hippocampal CRH neurons are engaged, as a first pass, we employed immunostaining of the immediate early gene c-fos, which is sharply upregulated and translocated to the nucleus following periods of heightened synaptic activity [33]. Starting from the basic assumption that a neuronal population’s relative synaptic activity increases during states or tasks to which it is functionally relevant, we tested the longstanding hypothesis that hippocampal CRH neurons are important for mediating the effects of stress on the hippocampus [34–36]. First as a positive control, kainic acid-induced seizures gave rise to extensive c-fos induction among CRH neurons (57.2 ± 15.1%) compared to saline-injected littermate controls (2.3 ± 1.0%), demonstrating that this approach can discriminate periods of high activity (Fig 2b). Unexpectedly, c-fos expression in this subset of hippocampal CRH neurons did not change after subjection to acute restraint stress (2.6 ± 2.6%) nor after chronic restraint stress (7.0 ± 4.4%), compared to minimally-handled littermate controls (6.9 ± 2.3%) (Fig 2b), in contrast to CRH neurons in the paraventricular nucleus (PVN), which are highly activated by stress [37]. An alternative stressor, foot-shock stress, gave rise to a marginal, non-significant trend toward increased c-fos induction (12.2 ± 1.5%) (Fig 2b) (n = 3–4 mice per experimental group). Collectively these data indicate that hippocampal CRH neurons exhibit low basal activity and are heavily recruited in hyperexcited network states but not in stress states.

### Validating cell type-specific manipulations of hippocampal CRH neurons

**Fig 3:**
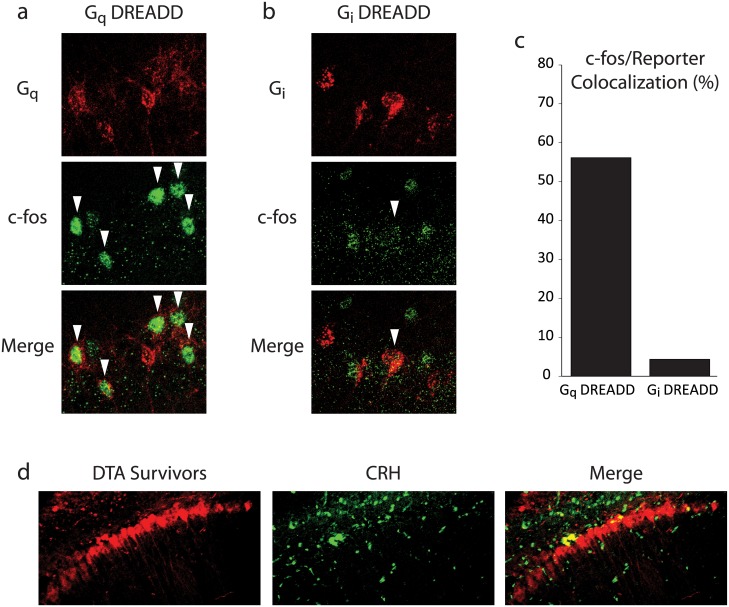
Validation of chemogenetic tools for selective activation, silencing, and ablation of hippocampal CRH neurons. a, Representative image of excitatory DREADD (G_q_) mCherry viral reporter expression (red) and c-fos immunoreactivity (green) in CA1 stratum pyramidale. White arrowheads indicate double-positive neurons. b, Representative image of inhibitory DREADD (G_i_) viral reporter expression (red) and c-fos immunoreactivity (green) in CA1 stratum pyramidale. c, The ratio of double-positive neurons to total reporter-positive neurons was determined. Chemogenetic stimulation (G_q_) broadly activates CRH neurons, while silencing (G_i_) effectively inhibits them. d, Representative image of AAV-FLEX-DTA expression in CA1 stratum pyramidale. Cre-negative pyramidal neurons transduced by the virus express mCherry (red) instead of DTA, marking the virus injection site. Diminished CRH immunoreactivity (green) around the injection site indicates that Cre-positive CRH neurons are effectively killed by DTA expression.

In order to further probe the functional relevance of this subset of hippocampal CRH neurons, we next employed chemogenetic tools to selectively manipulate these neurons *in vivo*. We began by validating these tools histologically. To selectively activate this population, BAC CRH-Cre mice were infused with a virus expressing excitatory (G_q_) Designer Receptors Exclusively Activated by Designer Drugs (DREADD). Hippocampal CRH neurons from these mice displayed a significant increase in c-fos expression (56.1%) 1 hr after i.p. CNO injection (Fig 3a and 3c). Conversely, BAC CRH-Cre mice infused with inhibitory (G_i_) DREADD-expressing virus showed minimal c-fos expression among hippocampal CRH neurons (4.3%) following CNO administration (Fig 3b and 3c) (n = 3 mice per experimental group). Finally, to irreversibly remove mature hippocampal CRH neurons from the network, we employed a virus that expresses fragment A of diphtheria toxin (DTA) in a Cre recombinase-dependent fashion, along with a Cre recombinase-independent red fluorescent reporter to mark adjacent surviving (i.e. Cre-negative) cells. To assess the loss of CRH neurons in the AAV-Flex-DTA animals, we compared the number of CRH-positive neurons throughout the CA1 region of the hippocampus in mice stereotaxically injected with AAV-Flex-DTA compared to AAV-GFP. Three weeks following viral injections, there is a 30.2 ± 3.3% reduction in the number of CRH-positive neurons in the CA1 region of the hippocampus (data not shown; n = 4–5 mice per experimental group). These data indicate that we can effectively express G_i_ and G_q_ DREADDs and AAV-Flex-DTA specifically in CRH neurons in the hippocampus.

The inhibition of hippocampal CRH neurons using G_i_ DREADDs was functionally confirmed using electrophysiological methods. The resting membrane potential of hippocampal CRH neurons expressing G_i_ DREADDs was decreased in the presence of CNO (-9.1 ± 4.0 mV). However, the addition of CNO did not significantly alter the resting membrane potential in hippocampal CRH neurons expressing only GFP (1.3 ± 0.5 mV) (S1 Fig; n = 7–8 cells, 4 mice per experimental group; p<0.05 using a paired Student’s t-test).

### Hippocampal CRH neuron activation suppresses excitability of the mossy fiber-CA3 pathway

**Fig 4:**
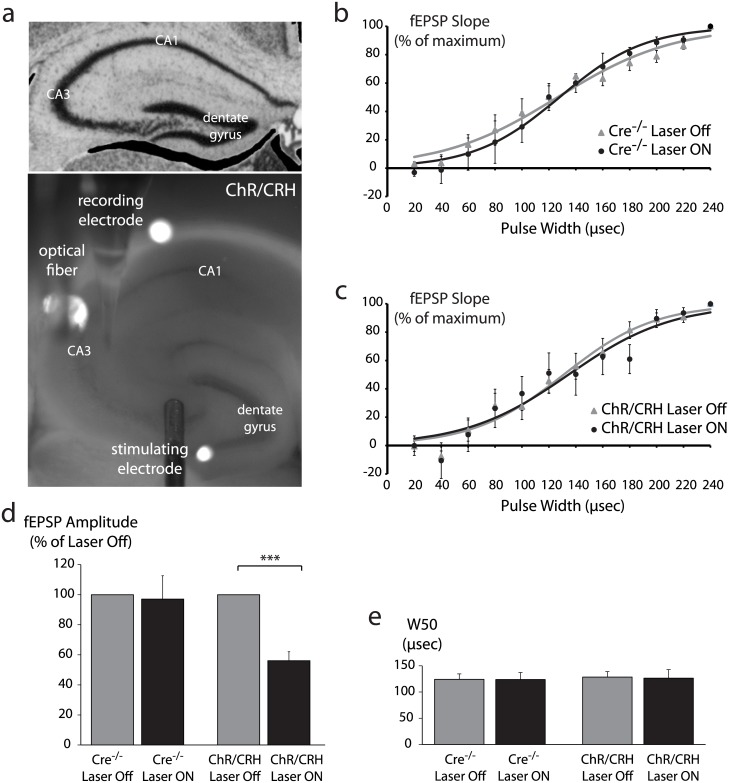
Optogenetic stimulation of hippocampal CRH neurons suppresses the excitability of the mossy fiber-CA3 pathway. a, Brightfield image of acute hippocampal slice from ChR/CRH mouse illustrates field potential recording approach. The stimulating electrode was placed over mossy fibers at the CA3-hilus border, while the recording electrode was tethered to an optical fiber and placed over CA3 *stratum radiatum*. b-c, Input-output curves generated from Cre^-/-^ control slices (b) and ChR/CRH slices (c) in the absence of laser (Laser Off) and during illumination by 473nm laser at 20 Hz (Laser ON). d, Laser illumination had no effect on peak fEPSP amplitude in Cre^-/-^ control slices, but substantially reduced peak fEPSP amplitude in ChR/CRH slices (p<0.001 by paired Student’s t-test). e, The pulse width required to evoke the half-maximal fEPSP response (W50) was derived from a Boltzmann function fit to the input-output data for each condition. In both Cre^-/-^ and ChR/CRH slices, laser illumination had no impact on W50.

We previously demonstrated that hippocampal CRH neurons back-project to form extensive inhibitory synapses on CA3 pyramidal neurons [12]. Therefore, we hypothesized that manipulating hippocampal CRH neurons’ synaptic output would impact the excitability of CA3. In order to test this hypothesis at the local network level, we employed field potential recording at the mossy fiber-CA3 synapse in conjunction with optogenetics (Fig 4a). Since this subset of hippocampal CRH neurons backprojects from CA1 to CA3, we tested the ability of optogenetic stimulation of the terminals onto CA3 pyramidal neurons to alter the population response to stimulation of the inputs onto these neurons coming from the dentate gyrus. Mossy fibers were electrically stimulated and fEPSPs were recorded in area CA3 to generate an input-output curve. After a 10 min recovery interval, a second input-output curve was generated while the hippocampal CRH terminals in area CA3 were illuminated by a 473nm laser (5msec pulses at 20 Hz) (Fig 4b and 4c). In slices from Cre^-/-^ mice, laser illumination had no impact on the half-maximal width of the input-output curve (baseline: 124.0 ± 10.6 μsec; laser on: 123.6 ± 13.5 μsec) or the peak fEPSP amplitude (97.0 ± 15.6% of baseline) (Fig 4d and 4e). In slices from ChR/CRH mice, laser illumination had no effect on half-maximal width (baseline: 128.4 ± 10.3 μsec; laser on: 126.2 ± 16.3 μsec) but strongly attenuated peak fEPSP amplitude (56.1 ± 6.0% of baseline) (Fig 4d and 4e) (n = 5–7 mice per experimental group; p<0.01 by paired Student’s t-test). These data demonstrate that hippocampal CRH neuron activation alone is sufficient to potently suppress the excitability of the mossy fiber-CA3 pathway.

### Hippocampal CRH neurons regulate locomotor activity and recognition memory

In light of their impact on the excitability of area CA3, we hypothesized that hippocampal CRH neurons play an integral role in hippocampus-dependent behaviors such as emotional behavior and learning and memory. To test this hypothesis we employed a battery of behavioral paradigms while selectively activating, silencing, or ablating hippocampal CRH neurons with excitatory DREADD (G_q_), inhibitory DREADD (G_i_), and DTA, respectively.

**Fig 5:**
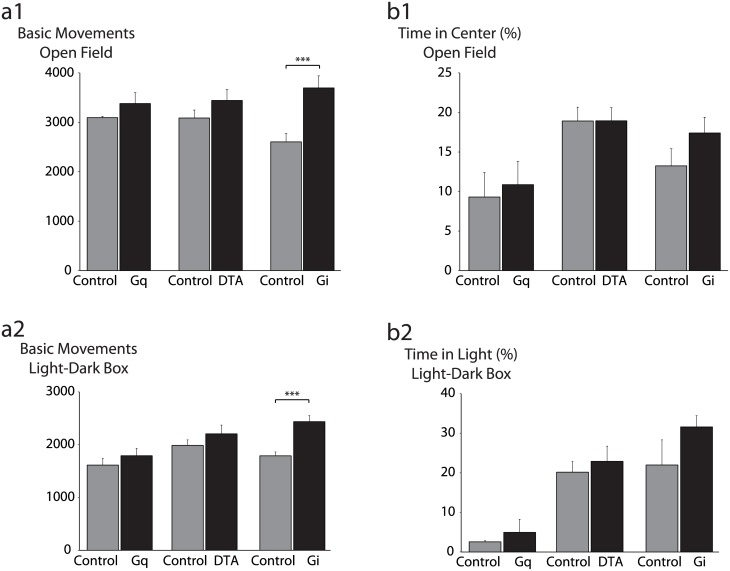
Impact of selective activation, silencing, and ablation of hippocampal CRH neurons on locomotor activity and anxiety-like behavior. a, Locomotor activity was assessed in the open field (a1) and light-dark box paradigms (a2). Neither chemogenetic stimulation (G_q_) nor ablation (DTA) significantly altered locomotor activity, while silencing (G_i_) gave rise to a significant locomotor hyperactivity phenotype in both the open field (p<0.005 by unpaired Student’s t-test) and light-dark box paradigms (p<0.001). b, Anxiety-like behavior was assessed in the open field (b1) and light-dark box paradigms (b2). Anxiety-like behavior was not significantly altered by any manipulation of hippocampal CRH neurons.

Selective activation of hippocampal CRH neurons by G_q_ did not alter locomotor activity (3379 ± 220 basic movements) or anxiety-like behavior in the open field (10.9 ± 3.0% time in center) compared to vehicle-injected controls (3095 ± 25 basic movements; 9.3 ± 3.1% time in center). Similarly, activation did not alter locomotor activity (1791 ± 139 basic movements) or anxiety-like behavior in the light-dark box (5.0 ± 3.3% time in light) compared to controls (1614 ± 126 basic movements; 2.6 ± 0.3% time in light) (Fig 5) (n = 12–17 mice per experimental group). Finally, selective activation had no impact on behavior in the forced swim test (latency to immobility: G_q_: 53.0 ± 15.3 s; Control: 65.0 ± 34.5 s; total time immobile: G_q_: 253.0 ± 8.1 s; Control: 234.0 ± 33.5 s) (S2 Fig).

Given our uncertainty over the relationship between the real time activity of hippocampal CRH neuron and physiological states (Fig 2), we next reasoned that silencing or ablating these neurons—effectively removing them from the hippocampal network—would be a more effective approach than activation for revealing their functional significance. Silencing CRH neurons using G_i_ did not significantly alter anxiety-like behavior in the open field (17.4 ± 2.0% time in center) or light-dark box (31.6 ± 2.8% time in light) compared to GFP-injected controls (13.2 ± 2.2% time in center; 22.0 ± 6.3% time in light) (Fig 5b). Surprisingly, silencing did produce a locomotor hyperactivity phenotype in both the open field (G_i_: 3696 ± 242 basic movements; Control: 2605 ± 169 basic movements; p<0.005 by Tukey’s HSD test) and light-dark box (G_i_: 2435 ± 115 basic movements; Control: 1788 ± 74 basic movements; p<0.001 by Tukey’s HSD test) (Fig 5a). Similarly, ablating CRH neurons with DTA did not impact anxiety-like behavior (DTA: 18.9 ± 1.7% time in center; Control: 18.9 ± 1.8% time in center), nor was locomotor activity in the open field test altered (DTA: 3441 ± 224 basic movements; Control: 3088 ± 161 basic movements) (Fig 5). Ablation of hippocampal CRH neurons did not significantly impact behavior in the light-dark box (22.9 ± 3.8% time in light; 2205 ± 167 basic movements) compared to controls (20.2 ± 2.7% time in light; 1987 ± 104 basic movements) (Fig 5). Depression-like behavior was also not significantly altered by silencing (G_i_: 194 ± 10 sec immobile; Control: 198 ± 9 sec immobile) or ablating CRH neurons (DTA: 209 ± 20 sec immobile; Control: 218 ± 16 sec immobile) (data not shown).

**Fig 6:**
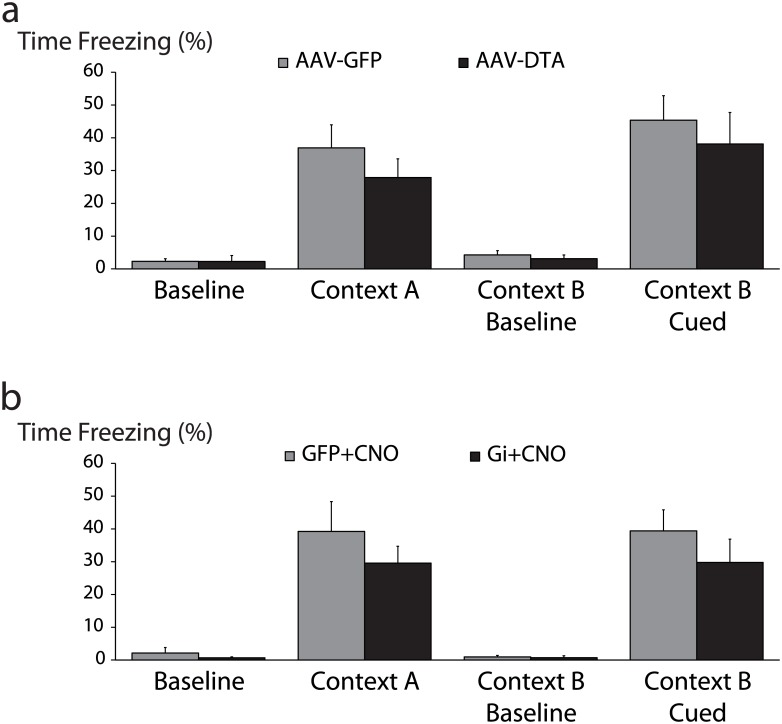
Selective silencing or ablation of hippocampal CRH neurons does not impact fear memory acquisition. a, Selective ablation of hippocampal CRH neurons (AAV-DTA) did not significantly alter fear memory retrieval either in the conditioned context (Context A) or in response to the conditioned cue (Context B Cued). b, Silencing hippocampal CRH neurons during acquisition (G_i_+CNO) had no impact on contextual or cued retrieval.

Cued fear memory acquisition, which is thought to be largely hippocampus-independent, was not significantly altered by silencing (G_i_: 28.1 ± 5.5% freezing; Control: 39.8 ± 5.3% freezing) or ablating hippocampal CRH neurons (DTA: 40.1 ± 6.4% freezing; Control: 34.7 ± 5.2% freezing) (Fig 6). Interestingly, hippocampus-dependent contextual fear memory acquisition was also intact after both silencing (G_i_: 25.8 ± 4.4% freezing; Control: 35.5 ± 7.4% freezing) and ablation of hippocampal CRH neurons (DTA: 24.2 ± 3.6% freezing; Control: 29.9 ± 4.7% freezing) (Fig 6) (n = 14–16 mice per experimental group).

**Fig 7:**
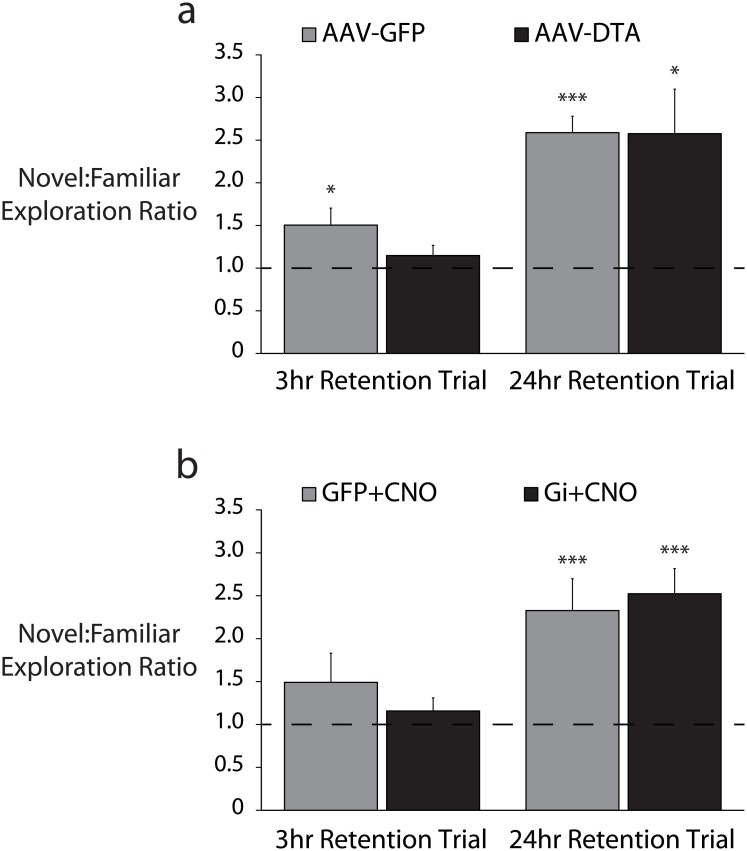
Selective silencing or ablation of hippocampal CRH neurons impairs object recognition memory. a, Following selective ablation of hippocampal CRH neurons (AAV-DTA), mice failed to preferentially explore a novel object 3hr after familiarization training (p = 0.24 by one sample Student’s t-test from a hypothetical mean exploration ratio of 1.00) while control (AAV-GFP) mice appropriately preferred the novel object (p<0.05). After a 24hr interval, both ablated and control mice appropriately preferred the novel object (p<0.05 and p<0.001 respectively). b, Hippocampal CRH neurons were selectively silenced during familiarization training (G_i_+CNO). After a 3hr retention interval, both silenced (G_i_+CNO) and control (GFP+CNO) mice failed to preferentially explore the novel object (p = 0.31 and p = 0.17 respectively). After a 24hr retention interval, both silenced and control mice appropriately preferred the novel object (p<0.001 and p<0.005 respectively).

Because silencing CRH neurons increased locomotor activity, it is possible that any changes in hippocampus-dependent fear memory acquisition were masked by hyperactivity. Therefore we decided to test recognition memory using a paradigm that internally controls for locomotor activity, the object recognition memory test [38]. In a retention trial 24 hours after familiarization training, neither silencing nor ablation of CRH neurons impaired mice’s ability to discriminate between a novel object and a familiar one (G_i_: 2.30 ± 0.36 novel:familiar ratio, p<0.05 from a hypothetical mean of 1.00 by one sample t-test; DTA: 2.58 ± 0.52 novel:familiar ratio, p<0.05 from a hypothetical mean of 1.00 by one sample t-test). However, after a 3 hour retention interval, mice whose CRH neurons were selectively ablated by DTA failed to discriminate between the novel and familiar objects (1.15 ± 0.12 novel:familiar ratio) (n = 12–16 mice per experimental group; p = 0.235 from a hypothetical mean of 1.00 by one sample t-test), in contrast to GFP-injected control littermates which preferentially explored the novel object (1.50 ± 0.20 novel:familiar ratio, p<0.05 from a hypothetical mean of 1.00 by one sample t-test) (Fig 7).

Together our behavior data indicate that CRH neurons of the dorsal hippocampus are important for modulating both locomotor activity and recognition memory.

### Hippocampal CRH neurons modulate the severity of kainic acid-induced seizures

**Fig 8:**
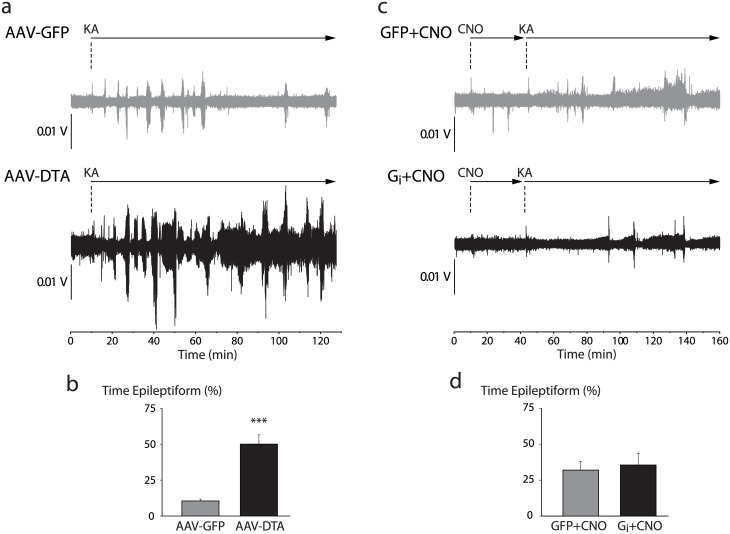
Selective ablation of hippocampal CRH neurons increases seizure susceptibility. a, Representative traces of EEG activity from control (AAV-GFP) and CRH neuron-ablated (AAV-DTA) mice demonstrating the induction of epileptiform and seizure activity by 10 mg/kg KA. b, Ablation of hippocampal CRH neurons significantly increased the total duration of KA-induced epileptiform activity compared to control mice (p<0.001 by unpaired Student’s t-test). c, Representative EEG traces from control (GFP+CNO) and CRH neuron-silenced (G_i_+CNO) mice demonstrating KA-induced epileptiform and seizure activity. d, Silencing hippocampal CRH neurons had no impact on the total duration of epileptiform activity compared to control mice (p = 0.75).

Ample evidence implicates abnormal hippocampal GABAergic signaling in the pathophysiology of temporal lobe epilepsy [39–41]. Additionally, our field recording experiments indicate that hippocampal CRH neurons exert a robust influence over the excitability of area CA3. Thus we hypothesized that manipulating hippocampal CRH neurons would be sufficient to modulate seizure susceptibility in the systemic KA model of temporal lobe epilepsy. Selective ablation of hippocampal CRH neurons by DTA gave rise to a profound increase in the proportion of time spent seizing (50.0 ± 6.4%) compared to GFP-injected controls (10.5 ± 1.3%; p<0.01 by unpaired Student’s t-test) (Fig 8a and 8b) (n = 6 mice per experimental group). Unexpectedly, selective silencing by G_i_ had no impact on seizure severity (G_i_+CNO: 35.5 ± 8.2%; GFP+CNO: 32.0 ± 6.1%; n.s. by unpaired Student’s t-test) (Fig 8c and 8d) (n = 7–9 mice per experimental group). The additional stress of CNO injection or off-target effects of CNO [18] in the latter experiment might have exacerbated seizure susceptibility (cf. Fig 8b AAV-GFP vs Fig 8d GFP+CNO), masking any proconvulsant effect of CRH neuron silencing. A more detailed analysis of electrographic seizure activity further revealed that mice receiving DTA spent significantly more time displaying high-frequency ictal discharges (34.2 ± 9.7%) than GFP-injected controls (8.9 ± 1.4%; p<0.05 by unpaired Student’s t-test) (data not shown).

### Model of hippocampal CRH neuron function

**Fig 9:**
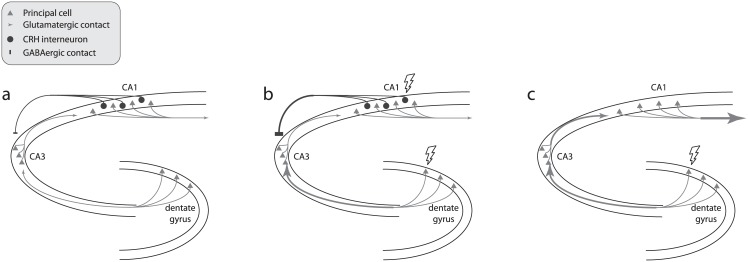
Hippocampal CRH neurons facilitate recognition memory and maintain adaptive network excitability by inhibiting CA3 pyramidal neurons. a-c, Simplified diagrams of the hippocampal trisynaptic circuit. Dentate gyrus granule cells (bottom right) project mossy fibers to CA3 pyramidal neurons (left), which project Schaffer collaterals to CA1 pyramidal neurons (top right), which in turn project to extrahippocampal brain regions. A subpopulation of CRH interneurons resides in CA1 *stratum pyramidale* and back-project to form inhibitory synapses on CA3 pyramidal neurons. a, Under basal physiological conditions, CRH neurons provide minimal inhibitory output to CA3. b, During periods of heightened hippocampal network activity such as active exploration or temporal lobe seizures, CRH neurons are moderately or heavily engaged to inhibit CA3, helping to constrain network excitability within an adaptive range. c, When CRH neurons are removed, the excitability of the network is less constrained, impairing recognition memory and diminishing seizure resistance.

Based on our experimental manipulations of CRH interneurons, we propose a model to describe CRH interneurons’ functional role within the hippocampal network. Basally (i.e. when input to the hippocampal network is within a “normal” range, CRH interneurons exhibit low levels of synaptic output (Fig 9a). During periods of relatively high activation of the hippocampal network (e.g. temporal lobe seizures, exploration of a novel context), hippocampal CRH neurons are recruited to increase their GABAergic output onto CA3 principal neurons (Fig 9b), thereby suppressing activation of CA3 by mossy fibers and restraining the final output from CA1 principal cells within an adaptive range. In the absence of CRH neuron activity (either through chemogenetic silencing or ablation, high hippocampal network activity goes unchecked, resulting in a high level of CA1 output (Fig 9c) which is (1) permissive of temporal lobe seizures and (2) outside the optimal range for learning and memory.

## Discussion

Selective silencing or ablation of hippocampal CRH neurons produced a deficit in recognition memory, locomotor hyperactivity, and hypersensitivity to kainic-acid induced seizures. Additionally, stimulation of hippocampal CRH neurons reduced the excitability of the mossy fiber-CA3 pathway. Taken together, these results suggest that hippocampal CRH neurons are critical for maintaining an adaptive range of excitability that permits normal recognition memory and experience-dependent plasticity, and especially for counterbalancing activation of the hippocampal network in hyperexcited states like seizures. Importantly, considering the abundance of hippocampal interneuron types and the relative sparsity of hippocampal CRH neurons, our results further indicate that the unique back-projecting connectivity of CRH neurons imbues them with a disproportionately strong influence on the excitability of the hippocampal network. This model of hippocampal CRH neuron function is meant as a starting point to invite specific testing and refinement through future experiments. However, it is important to note that we did not assess non-selective silencing or ablation of non-CRH neurons in the hippocampus which may also alter hippocampal network excitability and impact these behaviors and/or epileptiform activity as well.

### Hippocampal CRH neuron activity across physiological states

Although we demonstrated that hippocampal CRH neurons are recruited in the pathological state of acute seizures, we have yet to identify a physiological state in which this population is highly active. The main drawback of using c-fos immunoreactivity as an activity marker is its temporal resolution, as this approach offers only a static snapshot of all recently active neurons. Selective ablation or silencing of CRH neurons indicated that their activity as a population is importantly involved in the overall function of the trisynaptic circuit; on the other hand, only in the extreme circumstance of a KA-induced seizure did they show a population-level increase in c-fos expression.

### Dorsal and ventral hippocampus

Another major question we have yet to address is that of potentially separable functional roles for CRH neurons in the dorsal and ventral divisions of the hippocampus. In this study we focused on manipulating CRH neurons in the dorsal hippocampus *in vivo*, but through histology we observed similar anatomical distributions of CRH reporter-expressing cells and similar patterns of activity marker expression in both divisions. A substantial body of work indicates that the dorsal/posterior division of the hippocampus is involved in largely emotionally-neutral episodic memory processing and spatial navigation, while the ventral/anterior division is specialized for emotional information processing including some aspects of stress reactivity (for review see [42, 43]). Thus it is plausible that we observed effects on memory but not on stress-reactive emotional behavior precisely because our selective manipulation of CRH neurons focused solely on the dorsal subpopulation. It will be important in the future to test whether a functionally distinct subpopulation of CRH interneurons exists in each anatomical division of the hippocampus.

### GABAergic versus peptidergic signaling

The network excitability effects we observe after silencing and stimulating hippocampal CRH neurons are consistent with respectively lower and higher levels of GABA release onto CA3 principal cells. On the other hand, we cannot yet account for corresponding changes in hippocampal CRH receptor signaling, and a full treatment of hippocampal CRH neurons’ function must reconcile their otherwise contradictory inhibitory (synaptic) and excitatory (peptidergic) modes of transmission. An important obstacle to achieving this reconciliation is the dearth of prior research into the basic mechanism of peptide release, in contrast to the thoroughly-studied mechanism of synaptic vesicle exocytosis [44, 45]. It is generally understood that brief, local elevations of intracellular calcium are sufficient to trigger neurotransmitter release, whereas exocytosis of dense core vesicles containing neuropeptides and other high molecular weight signaling molecules requires more sustained, diffuse calcium influx [46]. Beyond this, little is yet known about the molecular mechanisms underlying dense core vesicle exocytosis, so for now the field relies on pharmacology of CRH receptors and direct application of exogenous CRH to infer its function [13, 34]. These approaches are beset with limitations: first, the CRH receptor has multiple endogenous cognate ligands with multiple regional sources [47]; second, the effective range of volume transmission for these ligands remains unknown, so distant brain regions cannot be ruled out as sources of hippocampal CRH; and third, the physiologically relevant concentrations of hippocampal CRH in various brain states have yet to be accurately measured. However, powerful novel approaches, such as Chinese hamster ovary “sniffer” cells in conjunction with slice electrophysiology, promise to shed light on peptidergic signaling in the future [48, 49].

In summary, future investigations into back-projection CRH interneurons should focus on disentangling their GABA-dependent *versus* CRH-dependent functions, identifying different functional roles for their dorsal *versus* ventral hippocampal sub-populations, and assessing how their activity changes as a function of physiological state. The same lines of inquiry are also worth following for other hippocampal interneuron classes, specifically those that express additional signaling molecules like somatostatin and are present in both the dorsal and ventral aspects of the hippocampus.

## Supporting information

S1 Fig Electrophysiological confirmation of chemogenetic silencing of hippocampal CRH neurons. a, A representative trace of the firing rate of a hippocampal CRH neuron expressing Gi DREADD before and after the addition of CNO (500nM). b, The average change in resting membrane potential in hippocampal CRH neurons expressing either AAV-GFP or Gi DREADD upon activation with CNO.
(EPS)

S2 Fig Activation of hippocampal CRH neurons does not alter depression-like behaviors. The average latency to immobility (a) and the total time spent immobile (b) in the forced swim test is not significantly different between CRH-Cre mice stereotaxically injected with either AAV-GFP or Gq DREADD.
(EPS)
